# Activated neutrophils: A next generation cellular immunotherapy

**DOI:** 10.1002/btm2.10704

**Published:** 2024-08-13

**Authors:** Ninad Kumbhojkar, Samir Mitragotri

**Affiliations:** ^1^ Harvard John A. Paulson School of Engineering and Applied Sciences Allston Massachusetts USA; ^2^ Wyss Institute for Biologically Inspired Engineering Boston Massachusetts USA

**Keywords:** cell therapy, ex vivo engineering, immunotherapy, neutrophils

## Abstract

Cell therapies are at the forefront of novel therapeutics. Neutrophils, despite being the most populous immune cells in human blood circulation, are not considered a viable option for cellular therapies because of their short lifespan and poor understanding of their role in the pathophysiology of various diseases. In inflammatory conditions, neutrophils exhibit an activated phenotype. Activation brings about significant changes to neutrophil biology such as increased lifespan, inflammatory cytokine secretion, and enhanced effector functions. Activated neutrophils also possess the potential to stimulate the downstream immune response and are described as essential effectors in the immune response to tumors. This makes activated neutrophils an interesting candidate for cell therapies. Here, we review the biology of activated neutrophils in detail. We discuss the different ways neutrophils can be activated and the effect they have on other immune cells for stimulation of downstream immune response. We review the conditions where activated neutrophil therapy can be therapeutically beneficial and discuss the challenges associated with their eventual translation. Overall, this review summarizes the current state of understanding of neutrophil‐based immunotherapies and their clinical potential.

**Translational Impact Statement**

The manuscript provides a summary of the current status of neutrophils as a cell therapy modality. In the world of cells therapies with complex supply chains and manufacturing, activated neutrophils can offer a relatively simpler clinical translation aided by allogeneic sourcing and a plethora of choices for activating stimuli. The review provides scientists and practitioners with a summary of the benefits and challenges associated with the use of neutrophils for cell therapy.

## 1 INTRODUCTION

Engineered immune cells are emerging as therapeutics with great potential for several disorders.^1^, ^2^, ^3^ The clinical success of CAR‐T cells has brought about a paradigm shift in the therapeutic landscape by demonstrating the curative abilities of cell therapies in diseases that were previously incurable.^4^ Additionally, modified stem cells, dendritic cells, NK cells, and macrophages are being widely explored in the clinic for a variety of diseases.^1^ Antigen‐specific cytotoxicity of CAR‐T cells,^4^ antigen‐independent toxicity of NK cells,^5^ multipotency and immunomodulatory properties of stem cells,^6^ phenotypic plasticity of macrophages,^7^ and professional antigen presentation by DCs^8^ offer specific advantages in the treatment of various disorders. Usually the cells, after harvesting from an autologous or allogeneic source and before the adoptive transfer, are modified ex vivo by engineering the phenotype and/or augmenting the natural functions for better therapeutic outcomes. These modifications include genetic engineering, cytokine pretreatment, and cellular hitchhiking of the supplementary therapeutics.^1^

While dendritic cells, NK cells, macrophages, and T cells dominate the clinical landscape of immune cell‐based therapeutics, these account for only 35% of the total immune cells in human blood circulation. Neutrophils, which account for over 50% of the immune cell population in circulation, are not the subject of any of the currently active cell therapy clinical trials. Neutrophils act as the first responders to the site of infection/inflammation and possess several effector functions such as phagocytosis, secretion of reactive oxygen species (ROS), proteolytic enzymes and neutrophils extracellular traps (NETs). However, neutrophils are usually overlooked as a potential cell therapy, given their short life span and poor understanding of their role in the pathophysiology of several disorders. However, in recent years, the role of neutrophils in the context of various diseases has been studied widely, and certain populations of neutrophils (e.g., N1 neutrophils in case of cancer) are reported to be therapeutically beneficial.^9^, ^10^

The human body produces 10^10^ to 10^11^ neutrophils per day under homeostatic conditions in a highly regulated process called granulopoiesis.^11^ Mature neutrophils, which are terminally differentiated, are released into the circulation from the bone marrow. Out of the entire neutrophil population, 1%–2% are estimated to be in circulation, whereas the rest are reserved in the bone marrow.^12^ Retention of neutrophils in the bone marrow is mediated by the interaction between C‐X‐C motif chemokine receptor 4 (CXCR4) on the surface of neutrophils and C‐X‐C Motif Chemokine Ligand 12 (CXCL12) on the surface of bone marrow stromal cells.^13^ The release of neutrophils from the bone marrow is encouraged by the C‐X‐C motif chemokine receptor 2 (CXCR2) signaling via C‐X‐C Motif Chemokine Ligand 1 (CXCL1) and C‐X‐C Motif Chemokine Ligand 2 (CXCL2) secreted by the endothelial cells.^9^ However, under inflammatory conditions, the fine balance between CXCR2 and CXCR4 is disturbed, leading to emergency granulopoiesis.^9^, ^14^

Among the salient characteristics of neutrophils are the immediate recruitment to the injury site and excellent phagocytic capabilities, thus rendering neutrophils a frontline defense against microbial infections. These abilities also make neutrophils an ideal cell‐based drug carrier.^15^ Neutrophils internalize drug‐loaded nanoparticles ex vivo and upon systemic administration, can deliver drugs preferentially to the sites of infection or inflammation. Several preclinical reports have explored the idea of utilizing the excellent trafficking capabilities of neutrophils for drug delivery to areas such as the brain that are challenging to access with traditional routes.^16^, ^17^, ^18^, ^19^

In disease conditions, neutrophils exhibit primed or activated phenotypes that possess the ability to modulate the downstream immune response.^20^ Phenotypic changes like the secretion of ROS, inflammatory cytokines, and crosstalk with other immune cells resulting from the activation of neutrophils play a vital role in the downstream immunomodulation, making them an important effector cells in conditions such as solid tumors. Activated neutrophils also exhibit prolonged lifespan, and improved anti‐microbial capabilities, making them a more potent cell therapy candidate compared to previously used naïve neutrophils. Additionally, the large number of neutrophils in peripheral blood along with amenability to allogeneic transfer due to the HLA‐independent mechanism of action and recent advances in the stem cell‐based manufacturing of neutrophils further makes a concrete case for translation into an off‐the‐shelf cell therapy.

In this article, we discuss, in detail, the hallmarks of neutrophil activation and the variety of stimuli by which it can be triggered. We then delve deeper into the immunologically relevant outcomes of the phenotypic changes and provide our perspective on the therapeutic potential of, and the advantages and challenges to the clinical translation of activated neutrophils as a cellular therapy.

## 2 NEUTROPHIL ACTIVATION AND ASSOCIATED CHANGES TO NEUTROPHIL BIOLOGY

**FIGURE 1:**
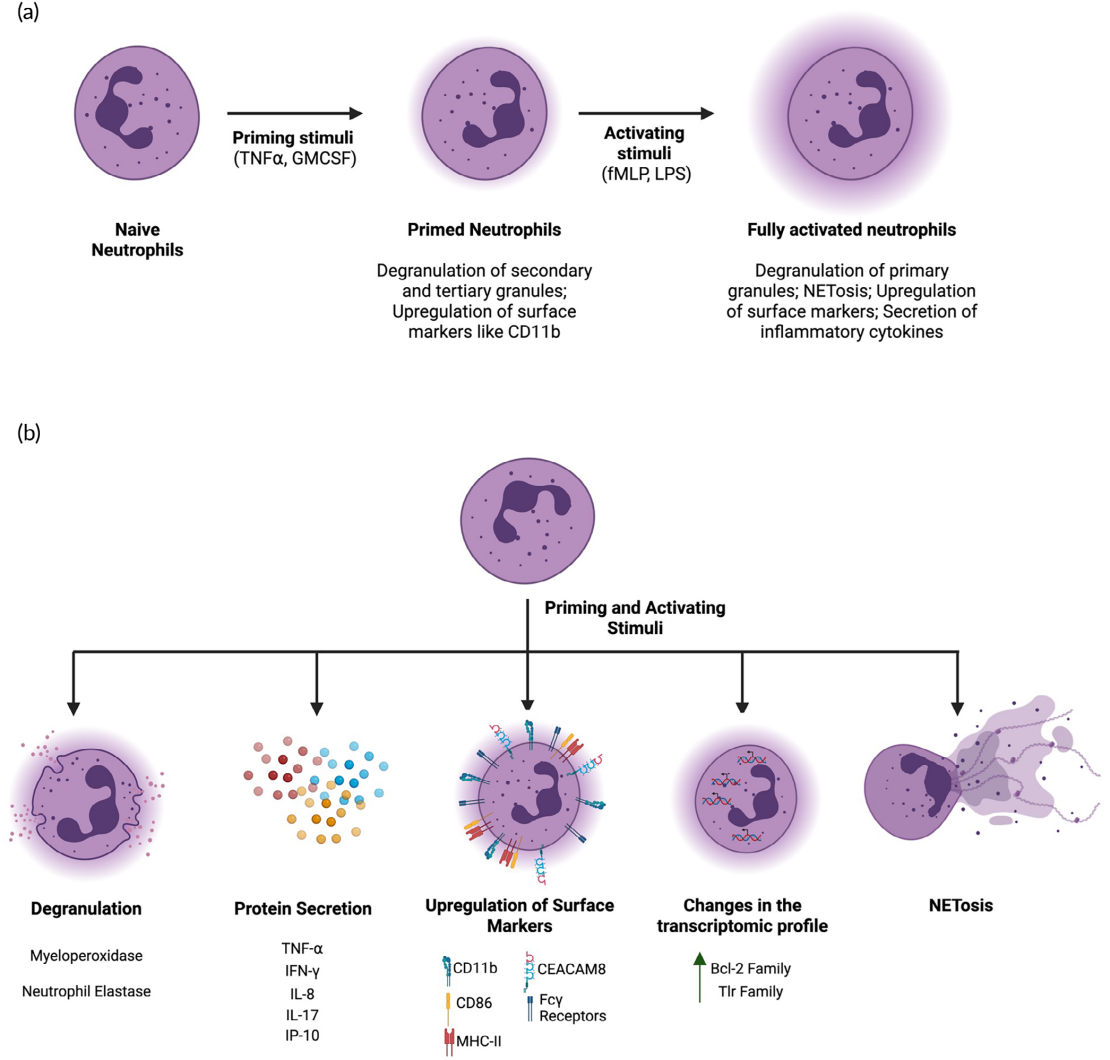
Activation leads to significant changes to neutrophil biology. (a) Neutrophils typically require two soluble stimuli for complete activation. Proinflammatory cytokines such as TNFα prime the neutrophils. Primed neutrophils secrete the contents of secondary and tertiary granules and change the surface marker expression. With another stimulus like fMLP or LPS, primed neutrophils change their phenotype to fully activated. Fully activated neutrophils secrete the contents of primary granules, upregulate surface markers such as Fc𝛾 receptors, antigen‐presenting signals (MHC‐II and costimulatory molecules), secrete pro‐inflammatory cytokines and undergo NETosis. (b) Activated neutrophils undergo degranulation, and secrete the contents of the granules, primarily MPO and NE. Neutrophils secrete various pro‐inflammatory cytokines. Activated upregulate various surface markers and alter the expression of several genes. Activated neutrophils also release the genetic material in their vicinity in the form of neutrophil extracellular traps (NETs). Schematic created using Biorender.com.

Activation of neutrophils is a two‐step process, consisting of priming or pre‐activation, and full activation.^21^ Priming allows neutrophils to respond to stimuli to become fully activated. The primary hallmark of neutrophil activation is degranulation. Neutrophils contain four types of granules; (i) azurophilic (or primary) granules, (ii) specific (or secondary) granules, (iii) gelatinase (or tertiary) granules, and (iv) secretory vesicles.^22^ During degranulation, granules fuse with the cell membrane, resulting in the release of the granule content and alteration in the expression of cell surface markers.^23^ Priming alone leads to the secretion of the contents of Specific and Gelatinase granules, whereas full activation is necessary for the release of the contents of azurophilic granules.^24^ Activation leads to significant changes to neutrophil biology, which are discussed in this section (Figure 1).

### 2.1 Release of proteins

Neutrophil granules are rich in a variety of proteins.^25^ Activated neutrophils release the contents of azurophilic granules, primarily myeloperoxidase (MPO) and neutrophil elastase (NE).^26^ MPO catalyzes the NADPH oxidase complex and promotes the production of hypochlorous acid from hydrogen peroxide.^27^ MPO is a primary mediator of the anti‐tumor activity of neutrophils.^28^ NE is an important mediator of host defense against pathogenic infection. Overproduction of NE can lead to host tissue damage.^29^ Activated neutrophils also release several proinflammatory cytokines and chemokines like TNF⍺, IFN𝛾, IL‐17, IP‐10, and IL‐8.^30^ Secretion of these cytokines may be a direct result of degranulation or overexpression of the pathways that produce them upon neutrophil activation.^31^ TNF⍺, IFN𝛾, and IL‐17 modulate the inflammatory phenotype of surrounding immune cells, whereas IL‐8 and IP‐10 are primary chemotactic agents for neutrophils and T cells respectively.^32^, ^33^, ^34^, ^35^

### 2.2 Changes in surface marker expression

Neutrophil activation alters the composition of cell surface proteins. Enhanced expression of MAC‐1 (CD11b/CD18) and CEACAM8 (CD66b) upon activation aids the firm attachment of neutrophils to the vascular surface during neutrophil extravasation.^36^ Activated neutrophils possess enhanced receptor‐mediated phagocytic capabilities by virtue of increased expression of Fc𝛾 receptors (CD16, CD32, and CD64) on the surface.^37^ Activated neutrophils exhibit an increase in the expression of MHC‐II and co‐stimulatory molecules like CD80 and CD86, rendering them potent antigen presenters.^38^ They possess the ability to internalize the antigen immune complexes and process and display epitopes on MHC‐II for the onset of antigen‐specific adaptive immune response.^39^

### 2.3 Altered gene expression

Resting neutrophils express the least number of genes related to translation among all leukocytes owing primarily to their short lifespan.^40^ However, upon activation, they are known to significantly alter their transcriptomic profile, leading to the overexpression of genes related to prolonged survival, enhanced antimicrobial activity and inflammatory cytokine secretion.^41^ Ericson et al. studied the gene expression of murine neutrophils treated with various stimuli (synovial fluid, uric acid and thioglycolate).^40^ Genes linked to the regulation of apoptosis (Bcl‐2 family), proinflammatory cytokine signaling via NFκB for responding to microbial products (Tlr family) and antigen processing/presentation were found to be highly upregulated. While all methods of activation led to the upregulation of these genes, interestingly, the extent of upregulation was varied. This can be a critical consideration when choosing the optimal method for the activation of neutrophils for eventual translation.

### 2.4 Formation of neutrophil extracellular traps (NETosis)

Neutrophil activation leads to the secretion of decondensed genetic material, termed neutrophil extracellular traps (NETs) in their vicinity in a process known as NETosis.^42^, ^43^ Following degranulation, various granule proteins such as MPO and NE leak into the cell, translocate into the nucleus, and aid chromatin decondensation by cleaving histones. Another nuclear enzyme, PAD‐4, catalyzes histone citrinullation. Decondensed DNA is then released into the cytoplasm.^44^ DNA is decorated with several neutrophil granule proteins such as MPO, NE, and PR3, among others before being released as NETs. Although the primary role of NETs is to aid microbial clearance, NETs also contribute to the modulation of immune response and pathogenesis of various diseases,^45^ which is discussed in the later sections of this article.

Activation of neutrophils, in vivo, is the first step of the cascade of immune response to an infection or injury. Activated neutrophils possess several characteristics that can be therapeutically beneficial or adversarial, depending on the type of ailment the patient is suffering from. With this consideration, we will discuss, in the following section, different methods of activation of neutrophils.

## 3 STRATEGIES TO ACTIVATE NEUTROPHILS

**FIGURE 2:**
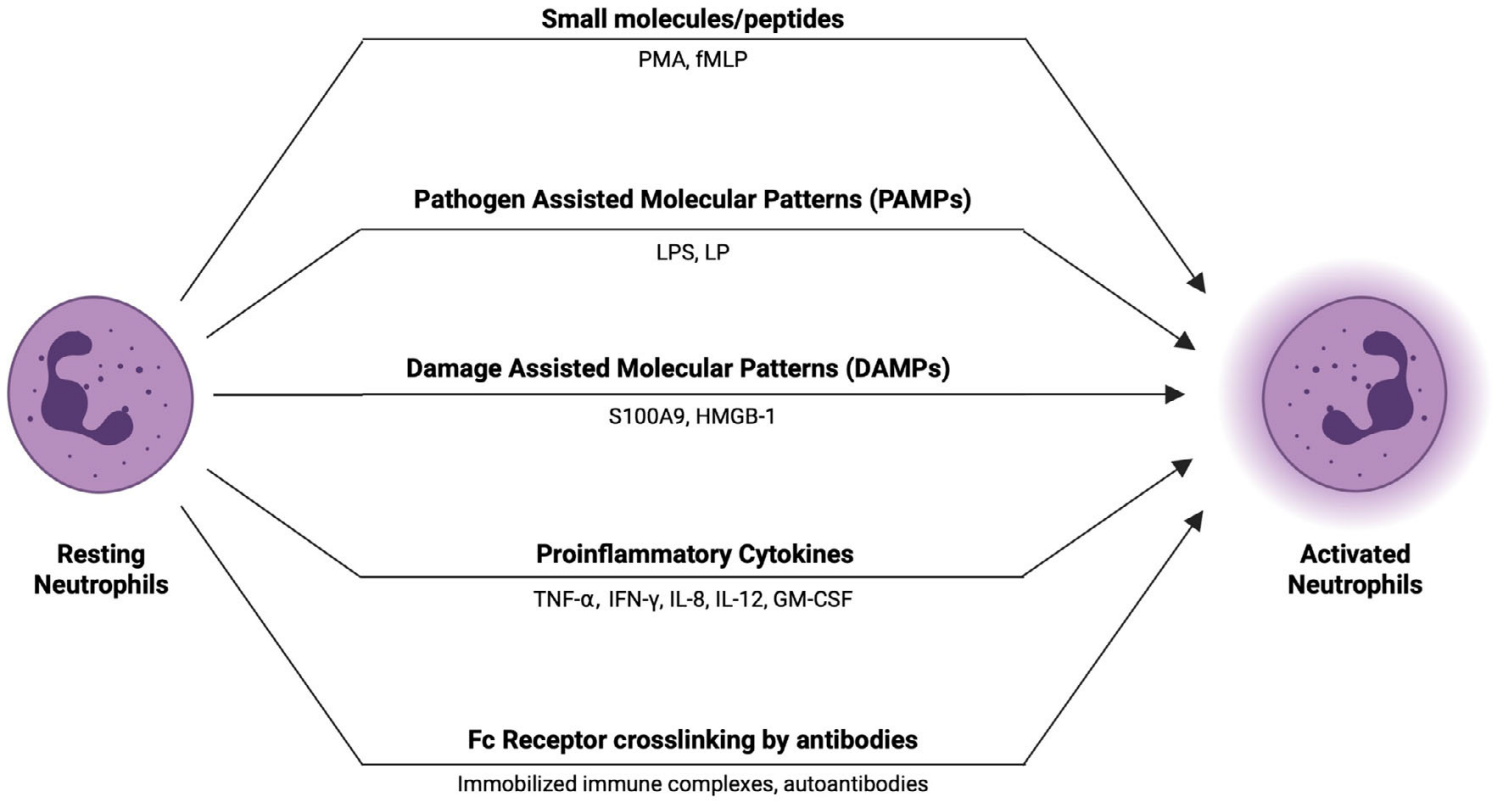
Neutrophils can be activated using variety of biochemical stimuli. Treatment with soluble factors such as small molecules, peptides, PAMPs, DAMPs, proinflammatory cytokines, and antibodies can potently activate neutrophils from their resting states. Schematics created using Biorender.com.

**FIGURE 3:**
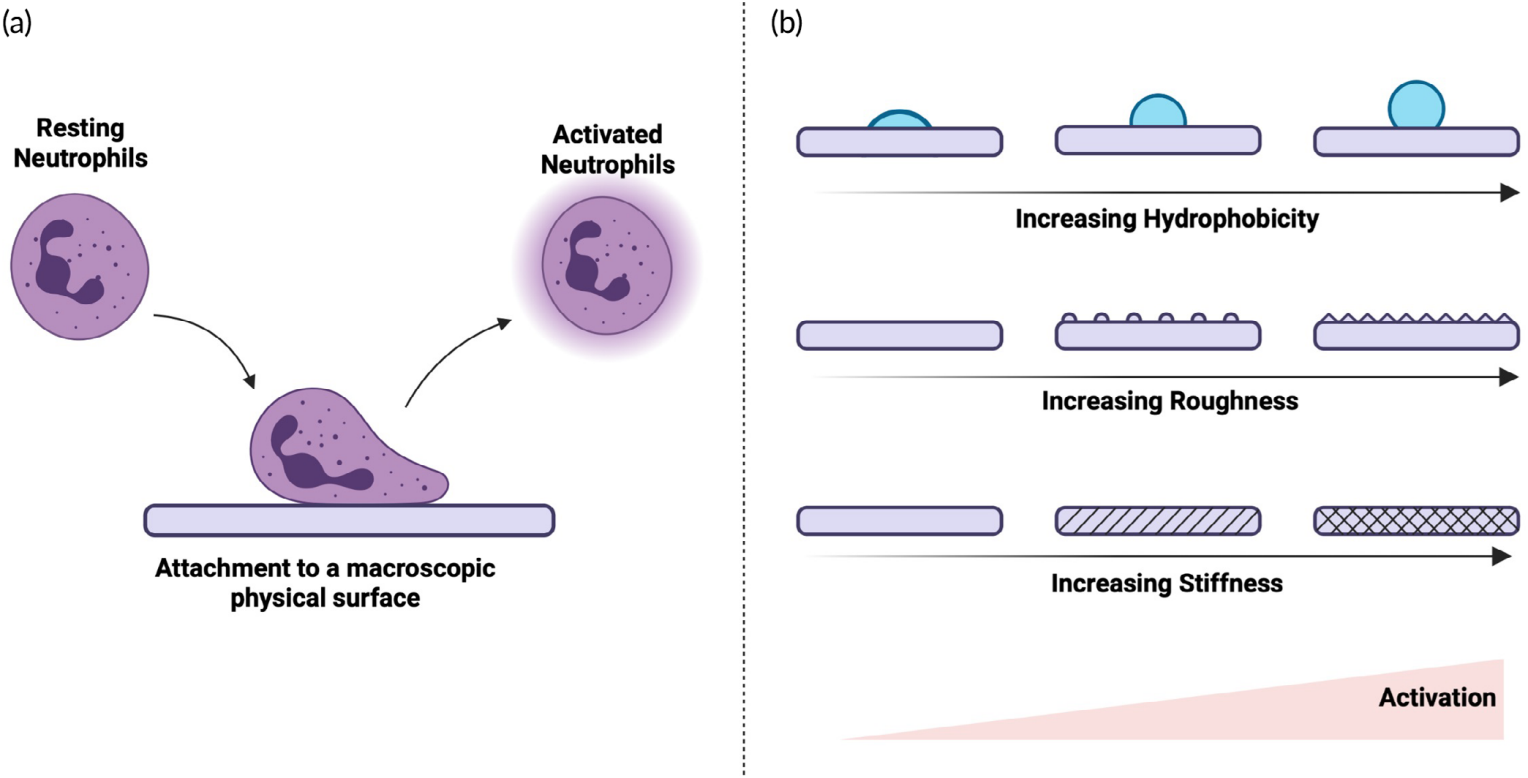
Physical ways of neutrophil activation. (a) Neutrophils activate when attached to a macroscopic surface. (b) The properties of the surface determine the magnitude of activation. Higher hydrophobicity, higher roughness, and higher stiffness lead to a higher activation. Schematics created using Biorender.com.

Neutrophils can be activated by several methods. We broadly classify these into two types: biochemical (Figure 2) and physical (Figure 3). Examples of biochemical ways involve the engagement of activating receptors and subsequent intracellular signaling primarily mediated by soluble factors like cytokines, pathogen‐associated or damage‐associated molecular patterns (PAMPs or DAMPs). Physical ways include the interaction of neutrophils with a surface whose properties regulate the extent of activation depending on their properties.

### 3.1 Biochemical ways of neutrophil activation

Small molecules like phorbol myristate acetate (PMA) and short peptides such as formyl‐methionyl‐leucyl‐phenylalanine (fMLP) have been used to study the biology of activated neutrophils for decades. PMA is known to enter the neutrophils and directly activate protein kinase C (PKC).^46^ PKC plays a vital role in the generation of ROS, cytoskeleton remodeling, and NETosis. In addition to its behavior as a chemoattractant for neutrophils, fMLP is known to activate neutrophils based on a MAP kinase‐dependent pathway mediated by the ligand–receptor interaction.^47^

Neutrophils are activated by PAMPs or DAMPs upon pathogenic infection or sterile injury. Pattern recognition receptors (PRRs), such as Toll‐like receptors (TLRs) present on the surface of neutrophils interact with the PAMPs resulting in neutrophil activation. PAMPs consist of microbial products such as lipoproteins (LP), lipopolysaccharides (LPS), or flagellin that lead to potent activation of neutrophils. Soler‐Rodriguez et al. studied the activation of neutrophils using LP and LPS in vitro and observed a dose‐dependent increase in the activation in the case of both components.^48^ LPS interacts with neutrophils via TLR‐4 leading to activation and increased survival. Dick et al. utilized dominant negative signaling constructs to elucidate the role of TLR‐4 in LPS‐mediated neutrophil survival.^49^ Neutrophils transduced with dominant negative TLR‐4, MyD88 or TRIF did not show an LPS‐dependent increase in survival, proving the role of TLR‐4/Myd88 pathway in LPS‐based neutrophil activation.

DAMPs such as S100A9 are highly prevalent in infection sites and in autoimmune conditions.^50^, ^51^ Simard et al. studied the effect on DAMPs on neutrophil activation in vitro.^52^ Interestingly, out of S100A8, A9 and A12, only S100A9 induced neutrophil degranulation in the MPAK‐Erk‐dependent manner. Treatment with S100A9 exhibited a dose‐dependent increase in ROS production, phagocytic and bactericidal activity mediated by the activation of Syk tyrosine kinases.^53^ High Mobility Group Box 1 (HMGB1) protein and histones are DAMPs released by stressed epithelial cells such as hepatocytes.^54^ Huang et al. reported that the NET formation by neutrophils was significantly increased upon treatment with the stressed‐hepatocyte‐conditioned medium.^55^ TLR‐4 and TLR‐9 KO neutrophils displayed significantly lower NET formation upon treatment with Histone or HMGB1, proving that the neutrophil activation by DAMPs can also be mediated by TLRs.

Upon exposure to PAMPs or DAMPs, tissue‐resident immune cells secrete inflammatory cytokines like TNF⍺, IFN𝛾, GMCSF, and neutrophil chemotactic agents such as IL‐8. These pro‐inflammatory factors are major contributors to the activation of neutrophils upon recruitment to the injured tissue. Different cytokines activate different intracellular signaling pathways. TNF⍺ acts via the activation of NFκB signaling, akin to LPS albeit resulting only in a primed phenotype rather than a fully activated one.^24^, ^56^ Neutrophils treated with TNF⍺ require another stimulus, such as fMLP, LPS, or antibodies for full activation.^24^, ^56^, ^57^ IFN𝛾 signaling is carried out via the JAK1‐Stat1 pathway, whereas GM‐CSF activates the Stat5 signaling pathways.^58^, ^59^ Neutrophils also respond to IL‐12 leading to a dose‐dependent increase in the secretion of IFN𝛾, in addition to increased phagocytosis, ROS production, and degranulation.^60^ IL‐12 further synergizes with LPS, IL‐15, and IL‐2 leading to a higher secretion of IFN𝛾. Moreno et al. demonstrated that neutrophil activation via IL‐12 and subsequent production of IFN𝛾 was critical in defense against polymicrobial sepsis.^61^

Several studies have also explored the role of antibodies in activating neutrophils. Antibody‐mediated activation is a result of the engagement of Fc receptors by surface crosslinking. The cross‐linking can be mediated by a combination of primary antibodies against the Fc receptors and secondary IgG against the Fc portion of the primary antibody, by immobilized immune complexes, or by autoantibodies against the neutrophil surface proteins. Crosslinking of Fc receptors is essential for the activation of neutrophils as the treatment with F(ab′)2 fragments does not elicit degranulation.^62^, ^63^ Fc𝛾RIIa and Fc𝛾RIIIb mediate the antibody‐dependent activation of human neutrophils whereas that of murine neutrophils is mediated by Fc𝛾RII and Fc𝛾RIV.^62^ Crosslinking of different Fc receptors leads to differential activation of neutrophils. Conflicting data exist on which Fc receptors are important for the activation of neutrophils. Jakus et al. showed that the increase in the ROS production and gelatinase release by neutrophils treated with immobilized immune complexes was mediated by both Fc𝛾RIIa and Fc𝛾RIIIb.^62^ Alemán et al. demonstrated that the crosslinking of Fc𝛾RIIIb and not Fc𝛾RIIa led to the activation and potent NETosis by human neutrophils.^64^ Teeling et al. showed that upon treatment with human IVIG, human neutrophils degranulate in a Fc𝛾RIIa‐, but not Fc𝛾RIIIb‐dependent manner.^65^ Autoantibodies, such as ANCA (antineutrophil cytoplasmic autoantibodies) generated in Wegener's disease, serve as the activating stimulus for the primed neutrophils mediated by Fc𝛾RIIa.^66^, ^67^ ANCAs are generated against proteins such as MPO and PR3 which are overexpressed by primed neutrophils. In the case of autoantibodies, crosslinking is achieved by the same antibody molecule binding to the antigen and Fc receptors on the same cell.

### 3.2 Physical ways of neutrophil activation

In addition to biochemical cues, neutrophils are also known to be activated upon attachment to synthetic physical materials (Figure 3a). Material properties, such as hydrophobicity, topography and stiffness are important in determining the level of neutrophil activation (Figure 3b). Material properties that can limit neutrophil activation and subsequent immune response are important design principles for medical implants. The immune system identifies medical implants (such as dental or orthopedic) as foreign objects and mounts an immune response to the site of implantation. Neutrophils being the first responders, accumulate at the site of the implant on a relatively quick timescale. Neutrophils try to destroy foreign objects by means of phagocytosis (if the size of the implant is amenable to phagocytosis) or secretion of factors that lead to proteolytic biodegradation.^68^, ^69^ To protect from biodegradation, implants are generally made from titanium. The surface properties of such titanium implants play an important role in the healing process of the site of implantation. Poor material choice (highly hydrophobic or rough surfaces) may lead to higher activation of neutrophils at the site of implants. NETs secreted by activated neutrophils may contribute to the formation of fibrous tissue,^70^ possibly impairing the medical implant's functionality. Pro‐inflammatory cytokines secreted from activated neutrophils may polarize macrophages to M1 phenotype^71^ instead of the desired M2 phenotype in case of wound healing, leading to improper wound healing and scar formation. Several reports have detailed the desired properties of the medical implants (stiffness, topography, hydrophobicity, etc.) that can limit or stimulate neutrophil activation. Drawing from these reports, following section summarizes the strategies that have been employed to potently activate neutrophils based on only physical stimuli.

Liu et al. demonstrated that the neutrophils attached to IgG‐coated hydrophobic glass surfaces released a higher amount of ROS than those attached to IgG‐coated hydrophilic glass surfaces, indicating higher activation in the case of hydrophobic surfaces.^72^ This activation was curtailed when the neutrophils were treated with Cytochalasin B which inhibits the actin filament function, indicating that the surface‐dependent activation is mediated by frustrated phagocytosis. Wetterö et al. further confirmed these observations.^73^ Sperling et al. tested the attachment of neutrophils to the surfaces of wide‐ranging water contact angles.^74^ Authors discovered that the neutrophils attached to hydrophobic surfaces such as Teflon AF™ were prone to increased NETosis compared to those attached to hydrophilic surfaces such as glass, despite 20‐fold higher attachment to glass compared to Teflon. Modification of hydrophobic surfaces with hydrophilic materials such as mucins resulted in reduced neutrophil activation.^75^ However, modification of surfaces with extracellular matrix proteins like Collagen, Fibronectin, Laminin, and Vitronectin led to higher neutrophil activation in a concentration‐dependent manner.^76^ This may be a result of the cross‐linking of adhesion ligands on neutrophil surfaces due to these ECM proteins.

Other surface properties such as topography are also known to play a vital role in determining the level of neutrophil activation with multiple studies reporting that surfaces with higher roughness lead to higher neutrophil activation. Chang et al. demonstrated that the neutrophils attached to roughened polystyrene released higher amounts of ROS compared to those attached to smooth polystyrene.^77^ Selders et al. demonstrated that the neutrophils treated with electrospun fibers of polydioxanone with 0.3 μm features (considered rough) led to higher amounts of NETosis compared to those with 1.9 μm features (considered smooth).^78^ However, conflicting results were reported by Abaricia et al.^79^ Authors demonstrated that the smooth hydrophobic titanium surfaces led to higher cytokine release, ROS secretion, and NETosis compared to rough hydrophobic surfaces. These contrasting results may be attributed to the varying properties of bulk materials used in each of these studies.

Neutrophils respond differently to the implantation of different biomaterials in vivo. Subcutaneous implantation of polystyrene microspheres led to higher ROS release from neutrophils compared to alginate microspheres.^69^, ^80^ NETosis was detectable on intraperitoneally implanted alginate microspheres in 7 days compared to 3 days for poly(methyl methacrylate) (PMMA) microspheres, indicating differential kinetics of neutrophil activation depending on the material properties. Interestingly, the bulk properties of materials such as stiffness also regulate the magnitude of neutrophil activation. Increasing stiffness leads to higher NETosis and higher secretion of proinflammatory cytokines.^76^ Abaricia et al. proved that the stiffness‐mediated activation is attributed to the focal adhesion kinase (FAK), a cytoplasmic tyrosine kinase that catalyzes the downstream integrin signaling.^76^

## 4 IMMUNE CELL CROSSTALK BY ACTIVATED NEUTROPHILS

By virtue of altered surface marker expression and protein release, activated neutrophils possess the ability to modulate the phenotype of surrounding immune cells. In this section, we summarize the latest advances in the understanding of these interactions between various immune cells and neutrophils.

### 4.1 Neutrophil‐dendritic cell crosstalk

**FIGURE 4:**
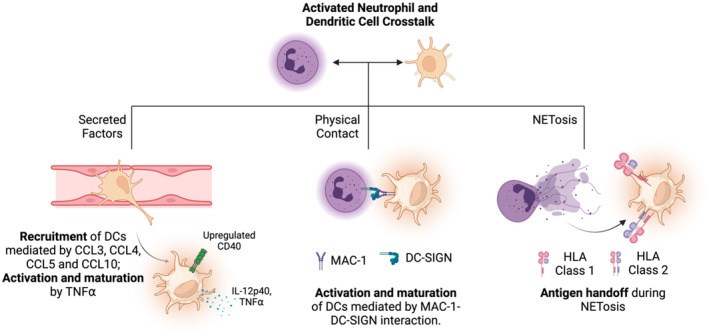
Crosstalk between neutrophils and dendritic cells. Activated neutrophils secrete factors that contribute to the recruitment and maturation of DCs. Direct physical interaction between MAC‐1 on neutrophils and DC‐SIGN on DCs contributes to potent activation and maturation of DCs. During NETosis, neutrophils may handoff phagocytosed antigen to DCs that can be displayed on HLA molecules for a downstream adoptive immune response. Schematic created using Biorender.com.

Dendritic cells (DCs), upon maturation and activation, are potent antigen‐presenting cells, that activate the adaptive immune response by co‐presenting danger signals (foreign antigen) and costimulatory signals to T cells. Activated neutrophils, by a variety of mechanisms, aid the augmentation of antigen presentation function of immature DCs (Figure 4).

Proteins released by neutrophils consist of a plethora of cytokines, chemokines, and alarmins that can recruit and activate the dendritic cells. Bennouna et al. demonstrated that the neutrophils activated by the treatment with *Toxoplasma gondii* secreted high amounts of chemotactic agents for immature DCs such as CCL3, CCL4, CCL5, and CCL10.^81^ Treatment of DCs with the supernatant of activated neutrophils increased the expression of CD40 on DCs confirming DC activation. The activation was partly mediated by TNF⍺ released by activated neutrophils as the inclusion of TNF⍺ blocking antibody curtailed DC activation. Additionally, activated neutrophils stimulated the DCs to secrete high amounts of IL‐12p(40) and TNF⍺. Park et al. demonstrated the importance of neutrophils‐DC crosstalk in vivo.^82^ Upon intrapulmonary challenge of Aspergillus, the DC migration from the lungs to the mediastinal lymph nodes was found to be impaired in neutropenic mice compared to non‐neutropenic mice. Lung resident DCs in the non‐neutropenic mice exhibited significantly higher maturation and costimulatory markers compared to those in the neutropenic mice. Degranulating neutrophils secrete alarmins such as defensins, lactoferrins, and cathelicidins that are known to activate and augment the antigen presentation function of immature DCs.^83^

In addition to the secreted factors, direct contact between activated neutrophils and DCs also plays a vital role in the crosstalk. van Gisbergen et al. proved that the neutrophils rapidly cluster with immature DCs when co‐cultured.^84^ This contact is mediated by the interaction between MAC‐1 overexpressed on activated neutrophils with DC‐SIGN (DC‐specific C‐type lectin) on immature DCs, leading to potent activation. The magnitude of activation (characterized by IL‐12p40 secretion by DCs), reduced considerably when neutrophils and DCs were separated by means of a transwell membrane indicating the importance of a physical cell‐to‐cell contact.^82^, ^84^ Megiovanni et al. demonstrated superior stimulation of T cells by DCs treated with activated neutrophils compared to naïve neutrophil‐treated or untreated DCs, indicating a functional benefit of activated neutrophil‐DC crosstalk.^85^

Activated neutrophils can hand antigens off to DCs via NETosis, which may lead to the pathogenesis of autoimmune conditions. Sangaletti et al. discovered that the DCs cocultured with NET‐forming neutrophils exhibited high levels of MPO and PR3, typical ANCA antigens.^86^ The authors showed that mice treated with NET‐loaded DCs had high levels of ANCAs in circulation and caused autoimmune renal damage. Diana et al. discovered that the NET‐forming neutrophils in the pancreas of young NOD mice release CRAMP (neutrophil granule protein cathelidin) that activates the production of IFN⍺ by the plasmacytoid DCs and initiated autoimmune diabetes.^87^

### 4.2 Neutrophil‐NK cell crosstalk

**FIGURE 5:**
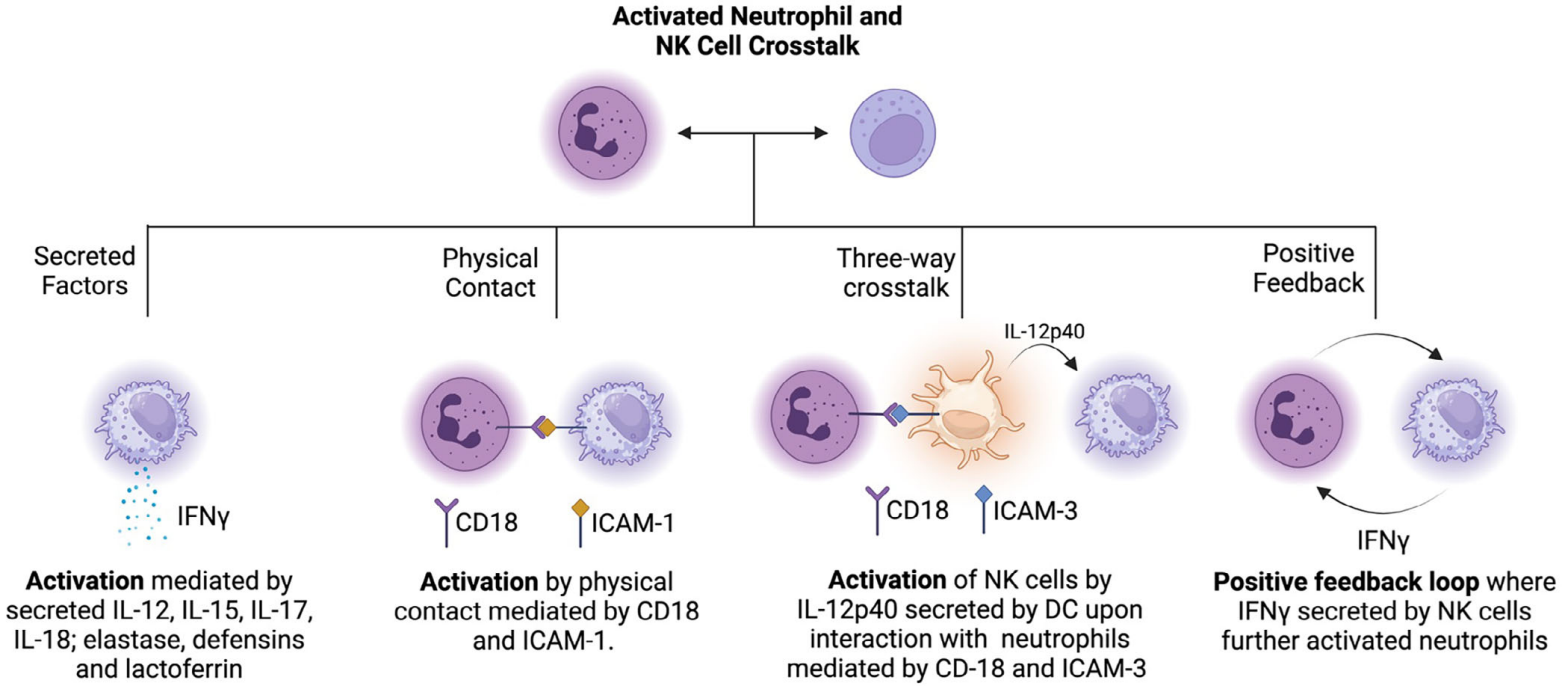
Interaction between NK cells and neutrophils. Pro‐inflammatory factors secreted by neutrophils directly activate NK cells in a paracrine fashion. Activated NK cells secrete IFN, that can further activate neutrophils in a positive feedback loop. Physical contact between neutrophils and NK cells mediated by CD18 and ICAM‐1 leads to potent activation of neutrophils. In an example of a 3‐way crosstalk, neutrophil can activate other immune cells like DCs in a receptor mediated fashion and the factors (like IL‐12p40) secreted by the third immune cells can activated neutrophils. Schematic created using Biorender.com.

NK cells are innate lymphoid cells that specialize in non‐specific toxicity towards malignant cells. Unlike T cells, NK cells exhibit cytotoxicity without antigen‐specific training from the APCs, making them a therapeutic of great interest for adoptive cell transfer.^88^ The cytotoxicity by activated NK cells is mediated by the direct contact with malignant cells via and subsequent release of granzymes and perforin or by antibody‐dependent cellular cytotoxicity.^5^ Activated NK cells produce high quantities of IFN𝛾, which leads to potent activation of surrounding immune cells.^89^ By various mechanisms, activated neutrophils are known to activate and augment the natural functions of NK cells (Figure 5). In this section, we will review the mechanisms and outcomes of the crosstalk between activated neutrophils and NK cells.

Jaeger et al. proved that neutrophils are vital in the functional development of NK cells.^90^ The authors observed that the NK cells from the patients with autoimmune neutropenia exhibited hyperproliferative phenotype and impaired cytotoxic function. Neutrophils seem to be especially important in the final stages of NK cell maturation in the lymphatic organs, where neutrophils and NK cells colocalize and form conjugates.^90^ Costantini et al. discovered that the direct contact between neutrophils and NK cells leads to higher secretion of IFN𝛾 from NK cells and is mediated by ICAM‐3 and CD18.^91^ NK cell activation by neutrophils is also known to be mediated by IL‐12p40‐producing SLAN+ DCs. Neutrophils stimulate the production of IL12p40, a stimulus for IFN𝛾 production by NK cells, by SLAN+DCs via CD18‐ICAM‐1 interaction.

Apart from direct cell–cell interactions, activated neutrophils can activate NK cells in a paracrine fashion. A variety of cytokines secreted by activated neutrophils including IL‐12, IL‐15, IL‐17, and IL‐18 are known to activate NK cells.^92^ Other neutrophil granule contents such as elastase, defensin, and lactoferrin also contribute to NK cell activation.^93^, ^94^, ^95^ However, some neutrophil products such as ROS and Arginase‐1 are known to curtail the effector function of NK cells.^96^ NK cell activation by neutrophils is a positive feedback loop wherein the IFN𝛾 produced by NK cells further activates neutrophils leading to increased activation and survival.^97^

### 4.3 Neutrophil‐T cell crosstalk

**FIGURE 6:**
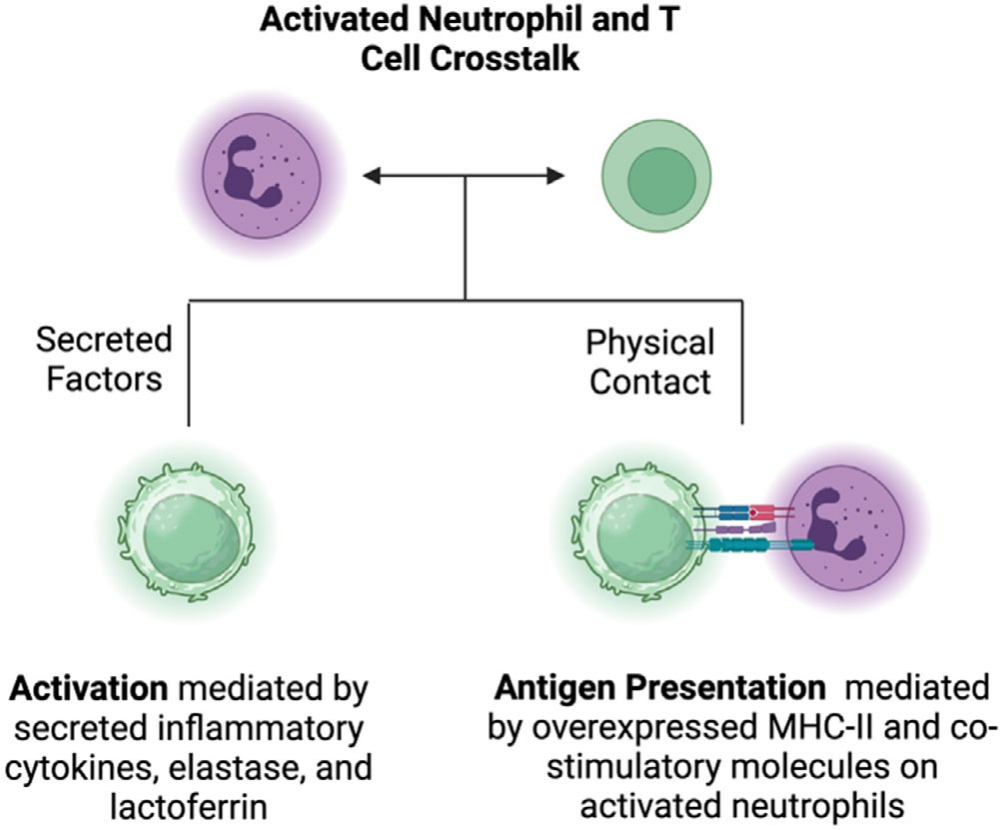
Interaction between neutrophils and T cells. Activated neutrophils secrete proinflammatory factors that can activate T cells in a paracrine fashion. Activated neutrophils overexpress MHC‐II and costimulatory molecules like CD80 and can serve as antigen presenting cells for a potent adaptive immune response by T cells. Schematic created using Biorender.com.

T cells form the backbone of the cell‐mediated arm of the adaptive immune system. T cells are trained by the antigen‐presenting cells to specifically target foreign antigens for the onset of an adaptive immune response. Activated neutrophils can modulate the phenotype and the function of T cells by means of paracrine signaling via secreted factors, or by direct cell‐to‐cell contact (Figure 6).

Several granule contents and cytokines secreted by activated neutrophils are known to directly suppress or activate the function of T cells. Inflammatory cytokines and granule contents such as elastase and lactoferrin enhance the activation and proliferation of T cells. Other granule contents such as serine proteases, Arginase‐1, and ROS inhibit or suppress the T cell activation. The fate of the interaction between the T cells and neutrophils depends on the activation status of both cells. Minns et al. demonstrated that the activated neutrophils and not resting neutrophils enhanced the activation, cytokine secretion, and proliferation in both CD4+ and CD8+ T cell subsets.^98^ However, the promotion of activating phenotype was most prominent for the T cells in an early activated phenotype. This observation is consistent with that of Oberg et al. Authors demonstrated that the activated neutrophils significantly enhanced the cytotoxic ability of short‐term expanded‐𝛾δ T cells while having a significantly lesser effect on the resting T cells.^99^

An important aspect of activated neutrophil‐T cell interaction comes from the APC‐like phenotype of activated neutrophils. Upon activation, neutrophils overexpress MHC‐II and other costimulatory molecules.^38^ Neutrophils can present antigens on MHC and subsequently activate the T‐cell immunity by direct cell‐to‐cell contact. In a seminal report, Mysore et al. demonstrated the change of neutrophil phenotype from the resting to antigen‐presenting DC‐like phenotype ex vivo.^100^ Authors observed that upon culturing neutrophils in the presence of GMCSF, an activating cytokine, and ovalbumin immune complex, neutrophils gained the features of DCs (CD11c, MHC‐II, CD80 expression, etc.) while maintaining the original features of neutrophils, making them a novel antigen‐presenting cell (termed nAPC) type. Authors demonstrated the functional benefit of the nAPCs ex vivo, using T cell proliferation assays and in multiple syngeneic tumor models in vivo.

## 5 ACTIVATED NEUTROPHILS AS A THERAPEUTIC

As discussed in the earlier sections, activated neutrophils produce primarily an inflammatory immune response. In many conditions, where chronic inflammation lies at the foundation of pathophysiology (COPD, autoimmune conditions such as rheumatoid arthritis, and multiple sclerosis, among others), treatment based on activated neutrophils may not lead to favorable therapeutic outcomes. However, the promise of activated neutrophils lies in the conditions in which the invigoration of the immune system is desired, such as cancer, where immunotherapeutics rely on switching the immunosuppressive (cold) microenvironment into the immunostimulatory (hot) microenvironment.

### 5.1 Cancer

Neutrophils are constantly recruited to the sites of solid tumors.^101^ Because of the highly immunosuppressive tumor immune microenvironment (TME), recruited neutrophils are polarized towards the N2 phenotype.^102^, ^103^ N2 polarized neutrophils elicit pro‐tumor functions such as ECM restructuring, angiogenesis and promote metastasis. Due to this, a high prevalence of tumor‐associated neutrophils (TANs) has been traditionally linked to a worse prognosis.^104^

**FIGURE 7:**
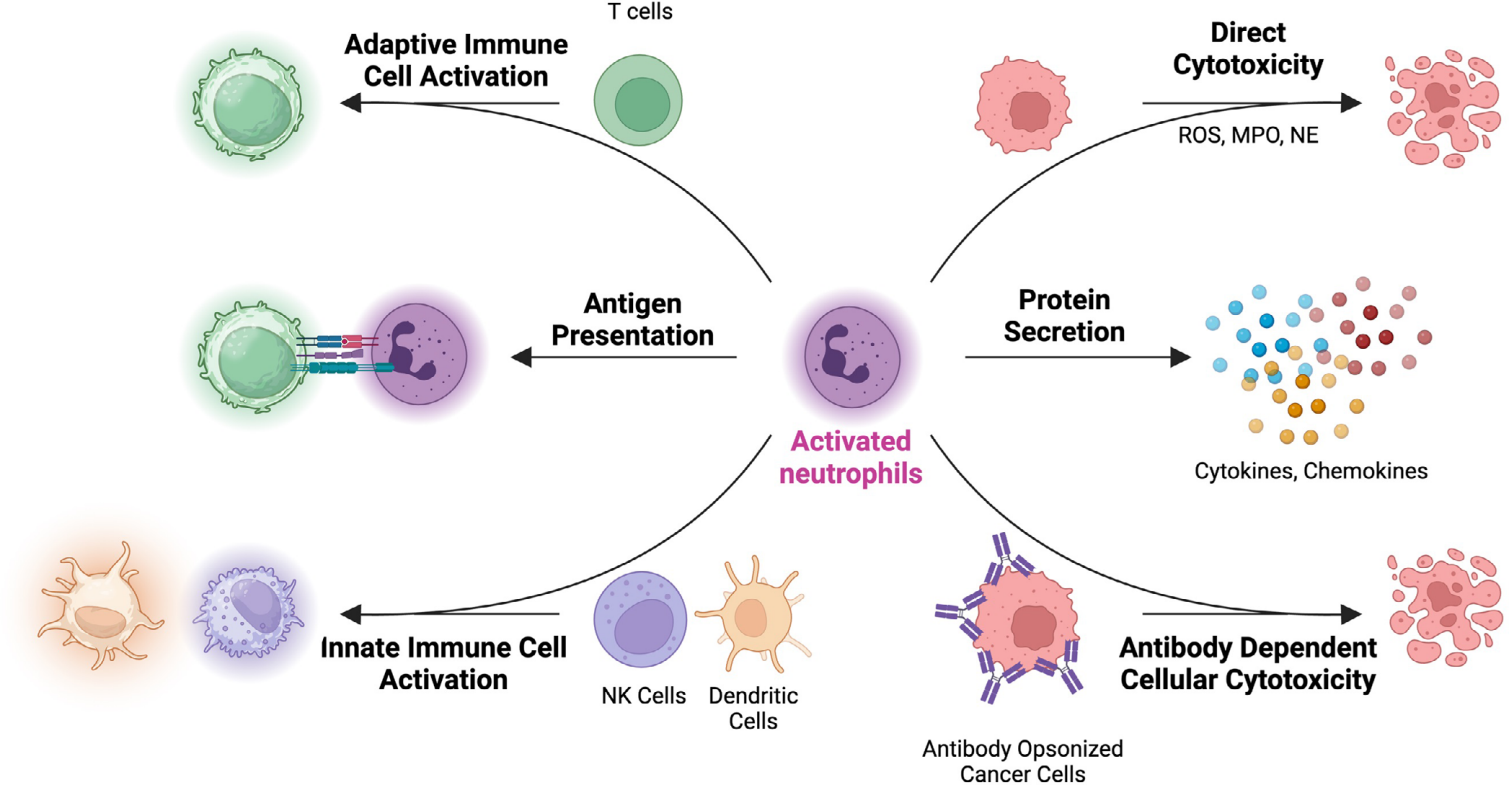
Activated neutrophils perform several anti‐tumor functions. Activated neutrophils induce direct cytotoxicity against cancer cells mediated by reactive oxygen species or by antibody‐dependent cellular cytotoxicity (ADCC). Activated neutrophils secrete various cytokines and chemokines that recruit and activate other immune cells. Via secreted cytokine or via direct cell‐to‐cell contact, activated neutrophils can activate the anti‐tumor phenotype of surrounding NK cells, dendritic cells, and T cells. Activated neutrophils can present antigens to T cells for the onset of an antigen‐specific immune response. Schematics created using Biorender.com.

However, a more nuanced role of neutrophils has come to light in the context of cancers in recent years. Neutrophils, in their activated phenotype, possess several anti‐tumor effector functions (Figure 7). Fridlender et al. reported that the presence of TGFβ in the tumor immune microenvironment polarizes neutrophils towards the N2 phenotype.^105^ Treatment with TGFβ inhibitor resulted in reduced tumor burden in a neutrophil‐dependent manner. The cytotoxicity towards tumor cells in the presence of TGFβ inhibitors was primarily due to activated neutrophils. The cytotoxicity was proved to be a result of the production of ROS, such as superoxides and hydrogen peroxide by activated neutrophils. This cytotoxic phenotype was defined as N1 in lieu of convention with tumor‐associated macrophages (M1 being anti‐tumor and M2 being pro‐tumor). In another report, Andzinski et al. demonstrated the ability of type 1 interferons, such as IFNβ, for N1 polarization of neutrophils in vivo.^106^ In fact, activated neutrophils have been discovered as primary effectors in response to immunotherapy. Hirschhorn et al. demonstrated that the recruitment of activated neutrophils to the tumors was important in the destruction of antigen‐loss tumor variants upon treatment with CD4+ T cells with OX‐40 co‐stimulation or CTLA‐4 checkpoint inhibition.^107^ Administration of a neutrophil‐activating cocktail (TNFα, anti‐OX‐40, and anti‐gp‐75 antibodies) into the tumors led to a complete regression. Linde et al. demonstrated that tumor regression is dependent on the recruitment of activated neutrophils.^108^

This phenotypic plasticity of neutrophils can be leveraged to develop a neutrophil‐based therapy for tumors. Ohms et al. reported a method to polarize neutrophils towards the N1 phenotype ex vivo with a cocktail of pro‐inflammatory cytokines.^109^ Authors observed that the N1‐like neutrophils demonstrate the cytokine release profile, increased antimicrobial capability and improved survival that is reminiscent of activated neutrophils. These data are in agreement with in vivo observations of Fridlender et al.^105^ From both these observations, it can be concluded that by activating the neutrophils ex vivo, it is possible to polarize them to an N1 phenotype. Adoptive transfer of these activated neutrophils holds the promise of being an effective cellular immunotherapy for cancer.

Adoptively transferred neutrophils are recruited to the injury or inflammatory sites such as the tumor on a relatively quick timescale.^16^, ^17^ Activated neutrophils can induce the antitumor effector function by both direct cytotoxicity and further modulation of the immune response (Figure 7). Production of ROS, MPO, and H_2_O_2_ mediate the direct cytotoxicity of tumor cells by neutrophils.^105^, ^110^, ^111^ Additionally, activated neutrophils possess the ability to elicit anti‐tumor effector function via antibody‐dependent cellular cytotoxicity (ADCC).^112^ Effector cells recognize the Fc portions of the antibody‐coated target cell.^113^ Once recognized, the target cells are lysed by the release of the contents of cytotoxic granules by the effector cells, phagocytosis, or trogoptosis depending on the type of immune cell.^114^, ^115^, ^116^ NK cells follow a granule‐dependent pathway, macrophages utilize the phagocytic pathway and activated neutrophils use trogoptosis. Trogoptosis is a type of non‐apoptotic cell death that is potentiated by close cell‐to‐cell contact between the antibody‐opsonized tumor cells and neutrophils.^116^ The ability of activated neutrophils to specifically lyse the antibody‐coated tumor cells makes a promising case for synergy between activated neutrophil therapy and monoclonal antibody therapy. Interestingly, the isotype of the monoclonal antibody also dictates the efficacy of ADCC. ADCC based on IgA antibodies has been proven to be more potent than that based on IgG antibodies.^117^, ^118^ Otten et al. showed that the immature neutrophils were able to trigger tumor killing via IgA FcR and not IgG FcR.^119^ Additionally, the blockade of myeloid checkpoints such as CD47‐SIRPa enhances IgA‐mediated tumor killing.^120^

Inflammatory cytokines secreted by activated neutrophils possess the ability to turn cold, immunosuppressive TME into a hot immunogenic TME by activating the anti‐tumor phenotype of surrounding immune cells. Additionally, systemically administered neutrophils can accumulate in the lymphoid organs such as the spleen,^19^ where they can further activate DCs, T cells, and NK cells for a lasting immune response. Activated neutrophils also possess antigen presentation capabilities, which can be leveraged for the onset of tumor‐specific adaptive immune response.^100^ Building on such a strategy, the accumulation of activated neutrophils carrying neoantigen peptides in lymphatic organs such as the spleen and lymph nodes can form the basis for a neutrophil‐based cancer vaccine.

Several reports have demonstrated the ability of neutrophils to be genetically engineered to express tumor‐specific CARs on their surface. Chang et al. utilized homology‐directed repair (HDR) mediated insertion of first‐generation CAR constructs into the AAVS1 locus of human pluripotent stem cells followed by chemical differentiation to generate anti‐glioblastoma (targeting CLTX) or anti‐prostate cancer (targeting PSMA) CAR‐neutrophils.^121^, ^122^, ^123^ CAR neutrophils showed excellent tumor killing in vitro. In orthotopic glioma models, although the treatment with CAR neutrophils alone showed an improvement in tumor growth kinetics, it did not result in a significant improvement in survival.^121^, ^122^ The same group leveraged the ability of neutrophils as drug carriers by combining CAR neutrophils with mesoporous silica nanoparticles loaded with hypoxia‐activated pro‐drug tirapazamine in a trojan horse system and showed a significant improvement in survival in mice bearing orthotopic glioblastoma tumors.^121^

The promise of activated neutrophils as anti‐cancer immunotherapy is not without limitations. NETs released by activated neutrophils play a vital role in promoting tumorigenesis by enhancing the invasiveness of tumor cells and increasing the chances for metastasis.^124^ NETs can form physical protection around cancer cells, which prevents their interaction with effector cells such as T and NK cells.^125^ Matrix metalloproteinases released by activated neutrophils are also known to promote ECM restructuring and neovascularization.^126^ While ROS secreted by the activated neutrophils can aid the direct killing of malignant cells, they also lead to suppression of the effector function of surrounding immune cells.

### 5.2 Severe neutropenia after myeloablation

Patients undergoing myeloablation are at risk of opportunistic pathogenic infections and sepsis due to severe neutropenia. Neutrophil or granulocyte transfusion therapies have been used in the clinic for such patients for decades, without consistently positive therapeutic outcomes.^127^, ^128^ Some of the successful reports of the use of granulocyte transfusion therapies precede the prevalence of potent antibiotics, antifungals, and growth factors that reduce the duration of neutropenia.^129^ However, in some severe cases such as excessively prolonged neutropenia or multi‐drug resistant pathogens, granulocyte transfusion therapies are still considered an option. Among other challenges such as patient selection and the type of pathogen being targeted, these therapies suffer from the low viability of neutrophils ex vivo and low persistence in vivo. These therapies usually include harvesting neutrophils from an allogeneic donor and direct transfusion into the patients without any ex vivo modification to the cells. Unlike resting neutrophils, activated neutrophils exhibit prolonged survival and enhanced antimicrobial and phagocytic capabilities as discussed earlier. Modification of this process to activate the neutrophils prior to the transfusion may alleviate the challenges associated with these therapies while increasing efficacy.

## 6 TRANSLATION OF ACTIVATED NEUTROPHIL‐BASED IMMUNOTHERAPIES AND CHALLENGES

While the promise of activated neutrophil‐based therapies is compelling, there remain several challenges for their translation to the clinic (Table 1). Firstly, the heterogeneity of the neutrophil population presents a significant challenge to the clinical translation of these therapies. One of the many parameters used to distinguish between the neutrophil phenotypes is the density of neutrophils leading to their differential accumulation in a density centrifugation column.^130^ High‐density neutrophils are known to be mature and have a lobular nucleus whereas low‐density neutrophils are immature and have a ring‐like nucleus. Each phenotype can present a different effector function. For instance, high‐density neutrophils are known to naturally possess an anti‐tumor phenotype, whereas low‐density neutrophils promote tumor progression.^130^, ^131^ Increased granulopoiesis during inflammation leads to the release of immature neutrophils into the circulation from the bone marrow.^132^, ^133^ The use of such neutrophils for granulocyte transfer therapies may lead to reduced effectiveness of planned therapy. Therefore, identifying the most potent subpopulations of neutrophils, based on the disease to be targeted is of pivotal importance.^134^ Using advanced transcriptomic and proteomic analysis may help inform the correct phenotypes in greater detail.

**TABLE 1:** Summary of challenges to the translation of neutrophil based cellular therapies, and potential solutions.

Challenge	Potential solutions
Neutrophil heterogeneity	Identification of populations of interest using advanced multi‐omic technologies Differentiation from stem cells using strict, optimized protocols for consistent populations
Large number of neutrophils required for a therapeutically relevant dose	Cell harvest from multiple donors Multiple aphereses per donor Enhancement in granulopoiesis in the donors by treatment with filgrastim and/or dexamethasone Differentiation from stem cells
Low viability of neutrophils ex vivo	Ex vivo priming of neutrophils with pro‐inflammatory cytokines to enhance the lifespan
Maintenance of activated phenotype in vivo	Supporting cytokine infusions that can provide continuous stimulations Development of neutrophils‐adhesive microscale materials that can provide activating stimuli to neutrophils under physiological stresses.

The other challenges faced by these treatments are the high number of cells required for transfusion and neutrophils' extremely short life span ex vivo. With decades of experience in granulocyte transfusion therapies, clinicians have concluded that for effective therapy, the minimum dose of neutrophils should be 0.6 × 10^9^/kg for one transfusion, with patients requiring multiple transfusions in most cases.^129^ Granulocyte transfusion for one patient usually requires apheresis from multiple donors or multiple aphereses per donor.^128^ Donors undergo a short‐term course of steroids such as dexamethasone and/or filgrastim (GCSF) to enhance the number of viable neutrophils in circulation.^128^, ^129^ However, such an artificial increase in the granulopoiesis may increase the fraction of immature neutrophils in the donors' blood circulation.

A way to tackle this manufacturing challenge is to utilize stem cells as a source of therapeutic neutrophils. Lieber et al. described a method to differentiate mouse embryonic stem cells into functional neutrophils.^135^ Yokoyama et al. described a method for the differentiation of human embryonic stem cells into functional neutrophils.^136^ Within 2 weeks in culture, more than 70% of the cells were differentiated into neutrophils. Nuclear morphology, surface markers and the function of neutrophils were tested and found to be similar to those of healthy peripheral blood neutrophils. Saeki et al. successfully demonstrated a feeder cell‐free approach to differentiate human embryonic stem cells into functional neutrophils to minimize contamination with animal‐derived factors.^137^ Morishima et al. described a method to differentiate human induced pluripotent stem cells (iPSCs) into functional neutrophils.^138^ LIfT Biosciences is utilizing a similar approach for manufacturing therapeutic neutrophils.^139^ LIfT Biosciences' N‐LIfT (Neutrophil based Leukocyte Infusion Therapy) platform is based on identifying the donors with a specific phenotype of neutrophils that possess excellent cancer‐killing abilities. Hematopoietic stem cells from such donors are then collected, expanded ex vivo and differentiated into functional neutrophils. The Bao group has demonstrated the ability to generation CAR‐neutrophils by chemically differentiating genetically modified hPSCs.^121^, ^122^ In addition to overcoming the scale‐up challenge, using stem cell‐based manufacturing techniques would also allow for greater control over the desired phenotype of neutrophils and the possibility to genetically engineer neutrophils to target specific malignancies.

Another challenge as mentioned above is maintaining the viability of neutrophils in vitro, prior to the transfusion. While the lifespan of neutrophils is a topic of debate because of a recent report by Pillay et al. which reports median lifespan of human neutrophils to be more than 5 days,^140^ it is a well‐accepted fact that neutrophils, especially in vitro, experience extremely short lifespan. However, activation of neutrophils prior to their use as a cell therapy can curtail this issue, as activated neutrophils are known to prolong their survival.^141^ Without activating stimuli, neutrophils undergo spontaneous apoptosis mediated by caspases. Pan‐caspase inhibitors have also been demonstrated to increase the lifespan of human neutrophils up to 48 h in vitro.^109^

The choice of method to activate neutrophils ex vivo and subsequent maintenance of the activated phenotype in vivo constitute critical parameters that determine the success of the clinical translation of this therapy. Different stimuli can bring about unique changes to neutrophil biology, which need to be thoroughly characterized prior to the selection. The timescale of stimulation required for the activation of neutrophils may vary for different stimuli. Additionally, although neutrophils undergo several phenotypic changes after activation, the exact sequence of those changes is not yet clearly studied. Some of the resulting phenotypes of activated neutrophils are desired over others (e.g., secretion of ROS and proinflammatory cytokines is desired over NETosis while treating cancer). Systematic investigations into the choice, quantity, and timescale of stimulation and resulting phenotype will help inform the choices of neutrophil activation for the condition being treated.

Each way presents a unique challenge to the translation as well. Several cell therapies currently being used in the clinic depend on the repeated dosage of supporting cytokines (e.g., IL‐2) for the maintenance of the desired phenotype.^1^ In a similar strategy, neutrophils activated in biochemical ways would necessitate supplementary doses of activating cytokines such as IFN𝛾, GMCSF or antibodies. The off‐target effects and toxicity of such supporting therapies would have to be considered while designing a therapy. In contrast, physical ways of activation would necessitate the development of micro‐scale materials that can be attached to and injected along with neutrophils in vivo. The material would have to be biodegradable for safe utility for humans. The choice of the materials should be guided by the properties that lead to robust activation of neutrophils (e.g., hydrophobicity, stiffness) as discussed in the earlier sections. The attachment of the material will have to be robust for it to be maintained throughout the circulation in the presence of high shear forces. We recently demonstrated the use of ‘Cyto‐adhesive micro‐patches (CAMPs)’ for neutrophil‐based tumor immunotherapy.^142^ CAMPs are discoidal particles that, upon attachment to neutrophils, remain on the surface of neutrophils without internalization owing to their geometry. CAMPs are synthesized from PLGA, a biodegradable, hydrophobic, and stiff material that is excellent for activating the anti‐tumor phenotype of neutrophils. Neutrophils attached to CAMPs elicited an anti‐tumor phenotype and led to a significant reduction in tumor burden in multiple syngeneic tumor models. Particles such as these CAMPs can be further modified to include other biochemical activation stimuli for a more robust neutrophil activation and eventual anti‐tumor phenotype change.^7^, ^143^, ^144^, ^145^, ^146^, ^147^

## 7 CONCLUSION

A plethora of changes to the neutrophil biology upon activation makes neutrophils important effectors in the modulation of immune response in a variety of diseases. Tapping into this natural ability of neutrophils by augmenting it ex vivo by means of exogenous stimuli can help neutrophils emerge as an effective next‐generation cell therapy. However, heterogeneity of the neutrophil phenotype introduces intricate nuances to the neutrophils' biology in the context of various disorders, which need to be studied in greater detail for the successful translation of these therapies to the clinic.

## AUTHOR CONTRIBUTIONS

**Ninad Kumbhojkar:** Conceptualization; data curation; formal analysis; visualization; writing – original draft. **Samir Mitragotri:** Conceptualization; supervision; funding acquisition; writing – review and editing.

## CONFLICT OF INTEREST STATEMENT

SM and NK are inventors on patent applications related to neutrophils (owned and managed by Harvard University). SM is a board member and shareholder of Hitch Bio, and a scientific advisor and shareholder of Asalyxa.

## Data Availability

Data sharing is not applicable to this article as no new data were collected.
